# Accelerated (45 mW/cm^2^) Transepithelial Corneal Cross-Linking for Progressive Keratoconus Patients: Long-Term Topographical and Clinical Outcomes

**DOI:** 10.3389/fmed.2020.00283

**Published:** 2020-06-19

**Authors:** Xiaoyu Zhang, Ling Sun, Mi Tian, Yang Shen, Meiyan Li, Jing Zhao, Xingtao Zhou

**Affiliations:** ^1^NHC Key Laboratory of Myopia, Fudan University, Shanghai, China; ^2^Department of Ophthalmology, The Eye and ENT Hospital of Fudan University, Shanghai, China; ^3^Shanghai Research Center of Ophthalmology and Optometry, Shanghai, China

**Keywords:** keratoconus, cross-linking, transepithelial, accelerated, topographical

## Abstract

**Aims:** We characterized long-term clinical outcomes of accelerated (45 mW/cm^2^) transepithelial corneal cross-linking (ATE-CXL) for the treatment of progressive keratoconus.

**Methods:** Forty-two eyes from 37 patients treated for keratoconus were observed. ATE-CXL was performed using riboflavin and pulsed-light ultraviolet treatment (45 mW/cm^2^, 7.2 J/cm^2^). Structural and functional measurements were made after 1 week and 1, 3, 6, 12, 24, 36, and 48 months subsequently. Corneal topographic parameters were observed using Scheimpflug topography (Pentacam software).

**Results:** Surgery was uneventful in all subjects. Mean uncorrected (UDVA) and corrected distance visual acuity (CDVA) (logMAR) were 0.99 ± 0.58 and 0.44 ± 0.27 (*P* = 0.022), 0.24 ± 0.29 and 0.27 ± 0.35 (*P* = 0.601), at baseline and last follow-up, respectively. The pre-operative mean maximum keratometry (Kmax) value was 57.29 ± 9.13 diopters (D), and the thinnest corneal thickness (TCT) was 456.21 ± 44.66 μm. Mean Kmax was 56.67 ± 9.36 D, 4 years post-operatively (*P* = 0.781). TCT changed to 453.17 ± 46.76 μm at 4 years post-operatively (*P* = 0.780). Multiple linear regression indicated that patients with thinner pre-operative TCT (≤ 450 μm) showed decreasing post-operative average keratometry (Kavg) and increasing post-operative TCT. Patients with posterior central elevation (PCE) >80 μm showed decreasing post-operative Kavg as well as post-operative PCE. No complications were observed during follow-up.

**Conclusion:** Stabilization after ATE-CXL was achieved for the treatment of keratoconus. The clinical efficacy of ATE-CXL in advanced keratoconus patients with thin corneal thickness and greater PCE will require further investigation.

## Introduction

Keratoconus is a progressive keratopathy characterized by protrusion of the cornea and subsequent stromal thinning, leading to visual deterioration. Since the disease manifests early and affects both eyes, keratoconus significantly impact patients' quality of life and increases economic burden. Conventional corneal collagen cross-linking (CXL) has been used clinically for keratoconus since 2003. This procedure consists of the removal of the central 9.0 mm-diameter corneal epithelium and instillation of riboflavin 0.1% drops for 30 min, followed by UV-A irradiation at 3 mW/cm^2^ for 30 min (1). It has been demonstrated to stop or slow the progression of corneal ectasia (2–4). However, debridement of epithelium by conventional CXL carries potential complications, including pain, difficulty in epithelial healing, stromal haze, sterile infiltrates, and keratitis (5, 6). Further, the required duration for the treatment can cause discomfort.

The original CXL procedure has been modified using different techniques to increase its efficacy. A transepithelial procedure was introduced to reduce patients' pain, accelerate visual recovery, and avoid the potential risks of epithelial removal (7, 8). Accelerated methods and modified treatment time based on the Bunsen-Roscoe law of reciprocity have come into use (9, 10). The accelerated transepithelial corneal collagen cross-linking (ATE-CXL) protocol preserves the epithelium, shortens the treatment duration by increasing the ultraviolet radiation power, and delivers the same total dose intensity as conventional CXL (11). Accelerated CXL protocols with UV-A intensity of 9, 18, and 30 mW/cm^2^ are currently most widely applied (12–14). In recent years, accelerated CXL using a UV-A intensity of 45 mW/cm^2^ has been suggested, with studies reporting favorable outcomes for the treatment of progressive keratoconus (11, 15). However, these studies are limited by a relatively short follow-up time, and long-term clinical outcome still requires investigation. Here, we observe the long-term clinical outcomes of ATE-CXL for keratoconus.

## Patients and Methods

### Subjects

This retrospective case series study was approved by the Ethics Committee of the Eye and ENT Hospital of Fudan University and adhered to the tenets of the Declaration of Helsinki. All participants were assessed to fulfill informed consent requirements. Forty-two eyes from 37 patients who were evaluated and treated for progressive keratoconus from December 2013 to 2014 were enrolled. The mean age of patients was 24.36 ± 4.17 years; 28 were male and 9 were female.

Inclusion criteria included a corneal topography map consistent with keratoconus (obtained by Pentacam; Oculus, Arlington, WA, USA), with an elevated posterior surface, corneal astigmatism increase of at least 1.00 diopter (D) or maximum cone apex curvature increase of at least 1.00 diopter (D) in the past year, and biomicroscopic signs. Exclusion criteria included patients with a history of corneal surgery, corneal pachymetry <380 mm as measured with a Pentacam, or pregnancy or lactation during the course of the study (16).

### Accelerated CXL (KXL System)

For the ATE-CXL procedure, patients were placed in a supine position and anesthetic eye drops were applied pre-operatively, after which a lid speculum was used. The corneal epithelium was left intact. Paracel (Avedro, Waltham, MA, USA, containing 0.25% riboflavin-5-phosphate) in corneal epithelial trephine (Model 52503B; 66 Vision-Tech, Suzhou, China) was used to cover the cornea completely for a total of 4 min. VibeX Xtra (Avedro, Waltham, MA, USA, containing 0.25% riboflavin-5-phosphate) was then used to rinse and cover the cornea with corneal epithelial trephine for a total of 6 min.

A KXL System (Avedro) was used to conduct ultraviolet treatment with pulsed illumination for 1 s at 45 mW/cm^2^, delivering a surface dose of 7.2 J/cm^2^. This treatment step lasted for 5 min and 20 s. A bandage contact lens was subsequently applied. Antibiotic drops were applied for 1 week, and topical steroids were administered for 16 days (four times a day initially, then reduced to once every 4 days).

### Ophthalmological Examination

Visual acuity (logMAR), slit-lamp examination, manifest refraction, Scheimpflug topography (Pentacam, Oculus Optikgeräte, Wetzlar, Germany), intraocular pressure (IOP), and endothelial cell density (ECD) were assessed in a pre-operative examination and 1 week post-operatively. Subsequent checks were made at 1, 3, 6, 12, 24, 36, and 48 months. Posterior central elevation (PCE) data were obtained from the corneal central point using Pentacam software. We applied same best-fit sphere (BFS) values to calculate PCE data across pre- and post-operative examinations at each follow-up, with ΔPCE defined by subtracting pre-operative from post-operative measures.

### Data Analyses and Statistical Evaluation

Data are expressed as means ± standard deviation (SD). We used one-way ANOVA for normally distributed continuous variables and a Kruskal–Wallis test for skewed continuous variables. Unadjusted multivariate linear regression analyses and corresponding 95% confidence intervals (CI) were calculated for the association between pre-operative and long-term post-operative average keratometry (Kavg), maximum keratometry (Kmax), TCT, and PCE data. Statistical analyses were performed using SPSS software, PASW 18.0 (SPSS, Chicago, IL, USA). *P*-values of < 0.05 were considered to indicate statistical significance.

## Results

All surgical procedures were completed successfully. No patients received additional surgery such as penetrating keratoplasty (PKP) for any reason. Among the 37 patients, five received ATE-CXL in both eyes. All patients completed the 4-year follow-up, with a mean follow-up time of 45 ± 4.89 months (longest follow-up 51 months). Baseline parameters are listed in Table 1.

**Table 1:** Pre-operation characteristics of enrolled keratoconus patients.

**Variable**	**Mean ± SD**	**Range**
Age (years)	24.36 ± 4.17	(18, 35)
Sphere (D)	−5.77 ± 4.69	(−17.5, 2.75)
Cylinder (D)	−3.01 ± 1.71	(−7.75, −0.25)
Spherical equivalent (D)	−6.96 ± 5.12	(−21.38, 0.75)
UDVA (logMAR)	0.99 ± 0.58	(0.10, 2)
CDVA (logMAR)	0.24 ± 0.29	(−0.18, 1.30)
Intracular pressure (mm Hg)	12.68 ± 3.62	(7, 20.9)
ECD (cells/mm^2^)	3,386.50 ± 365.16	(2,752, 4,065)
Kmax (D)	57.29 ± 9.13	(40, 79.5)
TCT (D)	456.21 ± 44.66	(380, 556)
PCE (μm)	58.69 ± 39.95	(3, 191)

*D, diopters; UDVA, uncorrected distance visual acuity; CDVA, corrected distance visual acuity; logMAR, logarithm of the minimal angle of resolution; ECD, endothelial cell density; Kmax, maximum Keratometry; TCT, thinnest corneal thickness; PCE, posterior central elevation*.

### Structural Analyses

#### Keratometry Values

**Figure 1:**
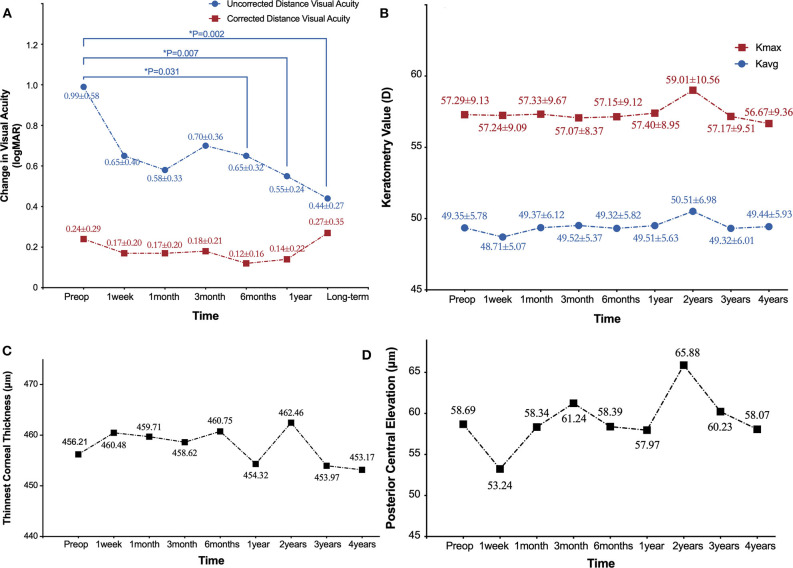
Refractive and topographical outcomes 4 years after ATE-CXL treatment. **(A)** Time course of change in visual acuity (logMAR). **(B)** Time course of change in average keratometry values (*K*avg) and maximum keratometry values (Kmax). **(C)** Time course of change in thinnest corneal thickness (μm). **(D)** Time course of change in posterior central elevation (μm).*indicates statistically significant difference from pre-operative measures.

**Figure 2:**
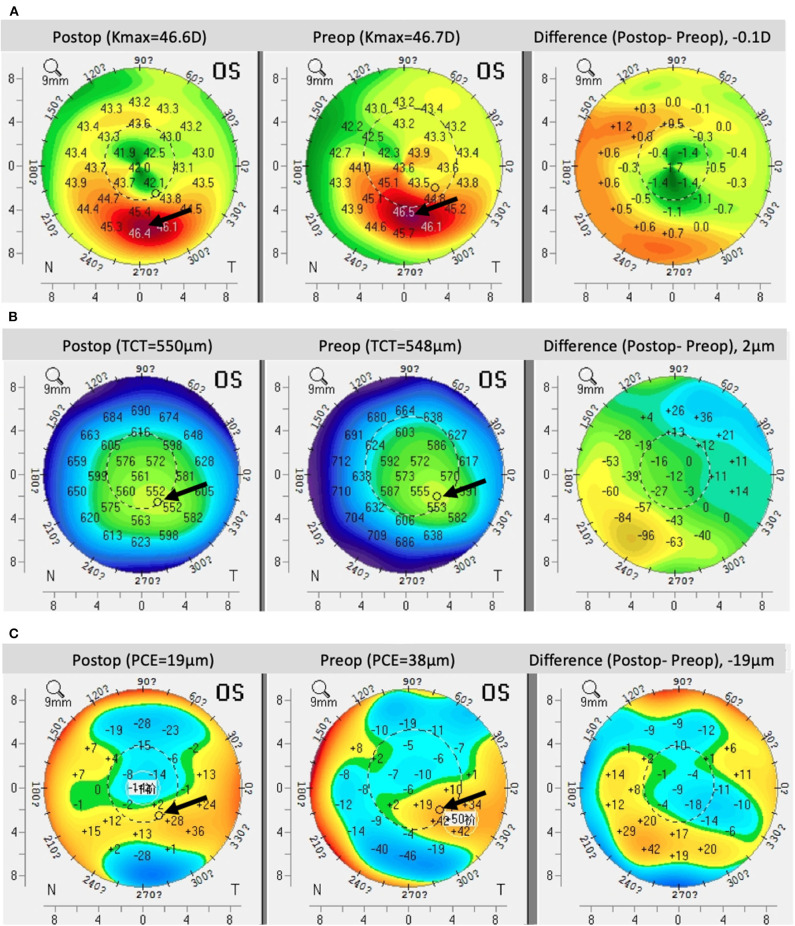
Pentacam imaging comparing **(A)** front curvature, **(B)** corneal thickness, and **(C)** posterior corneal elevation prior to treatment and 4 years after ATE-CXL treatment. Black arrows indicate the designated areas.

The mean steepest keratometry (Ksteepest), flattest keratometry (Kflattest), Kavg, astigmatism keratometry (Kast), and Kmax values were 50.74 ± 6.16 D, 47.97 ± 5.53 D, 49.35 ± 5.78 D, 2.77 ± 1.86 D, and 57.29 ± 9.13 D, respectively, at baseline (Table 2). At 48-months follow-up, the respective keratometry values were 50.65 ± 6.23 (*P* = 0.953), 48.23 ± 5.77 (*P* = 0.845), 49.44 ± 5.93 (*P* = 0.949), 2.42 ± 1.82 (*P* = 0.406), and 56.67 ± 9.36 (*P* = 0.781), with no significant differences arising during the follow-up period. Significant corneal flattening (Kmax ≥ 1.0 D) after 4 years occurred in 11 eyes (26.2%), and continued increase of ≥ 1.0 D in Kmax occurred in 11 eyes (26.2%). In cases with Kmax lower than 58 D, the mean pre-operative TCT was 491.90 μm and Kmax at 4 years increased by a mean of 0.14 D. Where Kmax was > 58 D, the mean pre-operative TCT was 439.38 μm, with a mean decrease after 4 years of 0.14 D (Figures 1, 2).

**Table 2:** Topography parameters, pre-operative and 1 Week, 1, 3, 6, 12, 24, 36, and 48 months post-operatively.

**Parameter**	**Pre-operative (*n* = 42)**	**Post-operative**							
		1 Week (*n* = 25) P^*^	1 Month (*n* = 35) P^*^	3 Months (*n* = 29) P^*^	6 Months (*n* = 36) P^*^	12 Months (*n* = 34) P^*^	24 Months (*n* = 26) P^*^	36 Months (*n* = 31) P^*^	48 Months (*n* = 30) P^*^
Ksteepest (D)	50.74 ± 6.16	50.03 ± 5.35 (*P* = 0.647)	50.73 ± 6.51 (*P* = 0.995)	50.79 ± 5.63 (*P* = 0.975)	50.59 ± 6.10 (*P* = 0.914)	50.83 ± 5.85 (*P* = 0.950)	51.84 ± 7.14 (*P* = 0.476)	50.56 ± 6.28 (*P* = 0.904)	50.65 ± 6.23 (*P* = 0.953)
Kflattest (D)	47.97 ± 5.53	47.39 ± 4.92 (*P* = 0.691)	48.02 ± 5.86 (*P* = 0.969)	48.25 ± 5.23 (*P* = 0.838)	48.05 ± 5.67 (*P* = 0.949)	48.20 ± 5.53 (*P* = 0.861)	49.18 ± 6.93 (*P* = 0.396)	48.07 ± 5.84 (*P* = 0.939)	48.23 ± 5.77 (*P* = 0.845)
Kavg(D)	49.35 ± 5.78	48.71 ± 5.07 (*P* = 0.665)	49.37 ± 6.12 (*P* = 0.988)	49.52 ± 5.37 (*P* = 0.908)	49.32 ± 5.82 (*P* = 0.980)	49.51 ± 5.63 (*P* = 0.906)	50.51 ± 6.98 (*P* = 0.431)	49.32 ± 6.01 (*P* = 0.979)	49.44 ± 5.93 (*P* = 0.949)
Kast(D)	2.77 ± 1.86	2.64 ± 1.69 (*P* = 0.759)	2.71 ± 1.94 (*P* = 0.884)	2.54 ± 1.70 (*P* = 0.583)	2.54 ± 1.78 (*P* = 0.561)	2.63 ± 1.71 (*P* = 0.730)	2.66 ± 1.72 (*P* = 0.800)	2.49 ± 1.69 (*P* = 0.506)	2.42 ± 1.82 (*P* = 0.406)
Kmax (D)	57.29 ± 9.13	57.24 ± 9.09 (*P* = 0.983)	57.33 ± 9.67 (*P* = 0.985)	57.07 ± 8.37 (*P* = 0.922)	57.15 ± 9.12 (*P* = 0.949)	57.40 ± 8.95 (*P* = 0.958)	59.01 ± 10.56 (*P* = 0.459)	57.17 ± 9.51 (*P* = 0.957)	56.67 ± 9.36 (*P* = 0.781)
Apex CT (μm)	465.64 ± 47.16	470.00 ± 44.56 (*P* = 0.718)	469.74 ± 50.99 (*P* = 0.708)	469.93 ± 46.48 (*P* = 0.710)	469.64 ± 47.86 (*P* = 0.713)	463.91 ± 49.36 (*P* = 0.875)	470.65 ± 49.76 (*P* = 0.675)	462.84 ± 44.83 (*P* = 0.804)	464.23 ± 47.80 (*P* = 0.902)
TCT (μm)	456.21 ± 44.66	460.48 ± 40.34 (*P* = 0.711)	459.71 ± 47.71 (*P* = 0.737)	458.62 ± 42.65 (*P* = 0.827)	460.75 ± 45.68 (*P* = 0.661)	454.32 ± 47.68 (*P* = 0.857)	462.46 ± 49.01 (*P* = 0.583)	453.97 ± 43.80 (*P* = 0.835)	453.17 ± 46.76 (*P* = 0.780)
PCE (μm)	58.69 ± 39.95	53.24 ± 30.82 (*P* = 0.569)	58.34 ± 35.83 (*P* = 0.968)	61.24 ± 36.08 (*P* = 0.780)	58.39 ± 35.69 (*P* = 0.972)	57.97 ± 37.42 (*P* = 0.934)	65.88 ± 43.39 (*P* = 0.447)	60.23 ± 40.82 (*P* = 0.864)	58.07 ± 38.53 (*P* = 0.945)
ΔPCE (μm)	0	0.96 ± 12.34 (*P* = 0.701)	2.49 ± 10.17 (*P* = 0.157)	2.48 ± 9.46 (*P* = 0.169)	1.31 ± 15.90 (*P* = 0.625)	−0.21 ± 14.95 (*P* = 0.936)	1.23 ± 16.88 (*P* = 0.713)	1.19 ± 17.75 (*P* = 0.711)	2.70 ± 21.62 (*P* = 0.499)

*D, diopters; Kavg, average Keratometry; Kcyl, cylinder Keratometry; Kast, astigmatism Keratometry; Kmax, maximum Keratometry; Apex CT, apex corneal thickness; TCT, thinnest corneal thickness; PCE, posterior central elevation; ΔPCE, PCE change between post-operative and pre-operative*.

* *compared with pre-operative*.

#### Pachymetry

Pre-operative apex corneal thickness (apex CT) and TCT were 465.64 ± 47.16 and 456.21 ± 44.66 μm, respectively. Apex CT was 463.91 ± 49.36 μm after 1 year and 464.23 ± 47.80 μm after 4 years (*P* = 0.875 and 0.902, respectively). TCT was 454.32 ± 47.68 μm after 1 year and 453.17 ± 46.76 μm after 4 years (*P* = 0.857 and 0.780, respectively; Table 2). At the end of the post-operative monitoring period, 40.5% of eyes showed no change or increase in TCT (Figures 1, 2).

#### Central Elevation Results

PCE values at the central corneal location changed from 58.69 ± 39.95 μm at baseline to 58.07 ± 38.53 μm after 4 years, but the difference was not statistically significant (*P* = 0.945). By the end of the monitoring period, 45.2% of eyes showed no change or reduce in PCE. Additionally, there was no significant difference between baseline and post-operative ΔPCE (Table 2; Figures 1, 2).

### Functional Analyses

#### Visual Acuity

At baseline, mean uncorrected distance visual acuity (UDVA) (logMAR) was 0.99 ± 0.58, and 0.65 ± 0.40, 0.58 ± 0.33, 0.70 ± 0.36 after 1 week, 1, and 3 months, respectively (all *P* > 0.05). After 6 months, there was a significant improvement in UDVA of 0.65 ± 0.32 (*P* = 0.031) compared to baseline. After 12 months, this improvement was 0.55 ± 0.24 (*P* = 0.001), and an improvement of 0.44 ± 0.27 (*P* = 0.022) was maintained at the end of the monitoring period. Pre-operative corrected distance visual acuity (CDVA) (logMAR) was 0.24 ± 0.29, 0.17 ± 0.20, 0.17 ± 0.20, 0.18 ± 0.21, 0.12 ± 0.16, 0.14 ± 0.22, and 0.27 ± 0.35 at 1 week, 1, 3, 6, 12 months, and at the end of the monitoring period, respectively (all *P* > 0.05). No change or gain in CDVA was recorded in 65.4% by the time of the final follow-up (Table 3; Figure 1).

**Table 3:** Functional analysis, pre-operative and 1 day, 1 week, 1, 3, 6, 12 months, and long-term post-operatively.

**Parameter**	**Pre-operative**	**Post-operative**					
		1 Week P^*^	1 Month P^*^	3 Months P^*^	6 Months P^*^	12 Months P^*^	Long-term P^*^
UDVA (logMAR)	0.99 ± 0.58	0.65 ± 0.40 (*P* = 0.059)	0.58 ± 0.33 (*P* = 0.017)	0.70 ± 0.36 (*P* = 0.073)	0.65 ± 0.32 (*P* = 0.031)	0.55 ± 0.24 (*P* = 0.007)	0.44 ± 0.27 (*P* = 0.002)
CDVA (logMAR)	0.24 ± 0.29	0.17 ± 0.20 (*P* = 0.361)	0.17 ± 0.20 (*P* = 0.308)	0.18 ± 0.21 (*P* = 0.387)	0.12 ± 0.16 (*P* = 0.066)	0.14 ± 0.22 (*P* = 0.225)	0.27 ± 0.35 (*P* = 0.679)
SE (D)	−6.96 ± 5.12	−6.15 ± 5.03 (*P* = 0.552)	−5.51 ± 4.64 (*P* = 0.270)	−5.12 ± 3.46 (*P* = 0.139)	−5.90 ± 2.10 (*P* = 0.052)	−5.82 ± 3.78 (*P* = 0.425)	−4.67 ± 5.40 (*P* = 0.055)
IOP (mmHg)	12.68 ± 3.62	12.24 ± 2.83 (*P* = 0.768)	12.94 ± 3.60 (*P* = 0.828)	10.81 ± 3.08 (*P* = 0.075)	10.95 ± 3.04 (*P* = 0.085)	12.36 ± 2.24 (*P* = 0.772)	12.34 ± 2.12 (*P* = 0.657)
ECD (cells/mm^2^)	3386.50 ± 365.16	3261.89 ± 560.88 (*P* = 0.733)	3279.20 ± 451.65 (*P* = 0.359)	3190.68 ± 396.62 (*P* = 0.051)	3207.10 ± 395.79 (*P* = 0.106)	3272.77 ± 330.73 (*P* = 0.341)	3266.88 ± 616.82 (*P* = 0.172)

*UDVA, uncorrected distance visual acuity; CDVA, corrected distance visual acuity; logMAR, logarithm of the minimal angle of resolution; SE, spherical equivalent; D, diopters; ECD, endothelial cell density*.

* *compared with pre-operative*.

#### Refraction

No statistically significant differences were observed in spherical equivalent (SE; D) during the post-operative follow-up (*P* > 0.05). The SE at baseline was −6.96 ± 5.12 D, and SEs at 1 week, 1, 3, 6, 12 months, and final observation were −6.15 ± 5.03, −5.51 ± 4.64, −5.12 ± 3.46, −5.90 ± 2.10, −5.82 ± 3.78, and −4.67 ± 5.40 D, respectively (Table 3).

#### Endothelial Cell Density

No statistically significant differences were observed in ECD (cells/mm^2^) during the post-operative follow-up compared to the pre-operative values (all *P* > 0.05). ECD at baseline was 3,386.50 ± 365.16 cells/mm^2^, and was 3,261.89 ± 560.88, 3,279.20 ± 451.65, 3,120.68 ± 396.62, 3,207.10 ± 395.79, 3,272.77 ± 330.73, and 3,166.88 ± 616.82 cells/mm^2^ at 1 week, 1, 3, 6, 12 months, and at the end of the monitoring period, respectively (Table 3).

#### Multivariate Linear Regression Analyses

Multivariate linear regression analyses suggested that when compared with patients whose pre-operative TCT was above 450 μm, patients with lower pre-operative TCT showed decreases of 1.17 D in post-operative Kavg value (*P* = 0.012, 95% CI = −2.03 to −0.30), and increases of 14.50 μm in post-operative TCT value (*P* = 0.009, 95% CI = 4.15–24.85) after 4 years (Table 4). When compared with patients whose pre-operative PCE was ≤ 80 μm, patients with greater PCE showed a decrease in post-operative Kavg of 1.53 D (*P* = 0.0058, 95% CI = −2.56 to −0.50) and a decrease in PCE of 23.59 μm (*P* = 0.0004, 95% CI = −35.57 to −11.60) after 4 years. There was no association between Kavg or Kmax and post-operative Kavg, Kmax, TCT, and PCE (Table 4).

**Table 4:** Multivariate linear regression analyses for effect of pre-operative data on long-term Kavg, Kmax, TCT, and PCE values.

	**Kavg**		**Kmax**		**TCT**		**PCE**	
	**(95%CI)**	***P***	**β (95%CI)**	***P***	**β (95%CI)**	***P***	**β (95%CI)**	***P***
Kavg > 48 D	0	–	0	–	0	–	0	–
Kavg ≤ 48 D	−0.39 (−1.31, 0.54)	0.4150	0.07 (−1.98, 2.12)	0.9495	−3.33 (−14.46, 7.79)	0.5603	−3.76 (−15.22, 7.70)	0.5237
Kmax > 55 D	0	–	0	–	0	–	0	–
Kmax ≤ 55 D	−0.45 (−1.38, 0.47)	0.3456	0.14 (−1.92, 2.20)	0.8960	5.01 (−6.11, 16.12)	0.3825	−5.98 (−17.40, 5.45)	0.3112
TCT > 450 μm	0	–	0	–	0	–	0	–
TCT ≤ 450 μm	−1.17 (−2.03, −0.30)	0.0120	−1.72 (−3.72, 0.29)	0.1010	14.50 (4.15, 24.85)	0.0090	−11.14 (−22.26, −0.02)	0.0565
PCE <80 μm	0	–	0	–	0	–	0	–
PCE ≥ 80 μm	−1.53 (−2.56, −0.50)	0.0058	−2.30 (−4.70, 0.09)	0.0670	13.09 (0.10, 26.08)	0.0552	−23.59 (−35.57, −11.60)	0.0004

*Kavg, average Keratometry; Kmax, maximum Keratometry; TCT, thinnest corneal thickness; PCE, posterior central elevation*.

#### Adverse Events

In all cases, slight stromal edema was noted soon after ATE-CXL procedures, but this resolved within 1 week. Complete epithelium was observed during all follow-up visits. No cases of infections or corneal melting were observed. Under slit lamp microscopy, no haze or corneal scars were noted.

## Discussion

Long-term clinical effects of ATE-CXL for the control of progressive keratoconus have not previously been systematically reported. Using Scheimpflug topography (keratometry values, pachymetry results), visual acuity, refractive results, and ECD, we demonstrated that better UDVA was achieved and sustained over 4 years of post-operative observation. We observed no statistically significant changes in keratometry, TCT, PCE, ECD, or refractive error values. Patients with thinner pre-operative TCT (≤ 450 μm) showed a decrease in post-operative Kavg value and an increase in post-operative TCT compared to patients with pre-operative TCT > 450 μm. When compared to pre-operative patients with PCE ≤ 80 μm, patients with PCE > 80 μm showed decreasing post-operative Kavg and PCE.

Since its introduction by Spoerl et al. (17) corneal collagen cross-linking has been described as the most promising innovation in the control of progressive keratoconus. This approach increases the bonds between collagen chains of the corneal stroma and improves the mechanical properties of the cornea. The long duration and epithelial debridement of traditional CXL procedures are believed to yield greater cross-linking effects and prolong the recovery of the cornea. Due to adverse events reported in previous studies (5, 6) accelerated CXL was introduced to intensify treatments while reducing exposure time, thereby also reducing the duration of surgery. Accelerated CXL protocols using 9 mW/cm^2^ (UV-A irradiation for 10 min), 18 mW/cm^2^ (UV-A irradiation for 5 min), and 30 mW/cm^2^ (UV-A irradiation for 3 min) are now widely used (18–20). Increased patient comfort and reduced risk of corneal dehydration improve the practical efficacy of this procedure. Higher-intensity UV-A irradiation (at 45 mW/cm^2^) has also recently been reported and appears to be safe and efficacious based on observations of keratoconus stability (11, 21). Therefore, we used transepithelial high-intensity CXL at 45 mW/cm^2^ and 7.2 J/cm^2^ using pulsed irradiation.

We observed a significant improve in UDVA after 4 years, as well as statistically stable CDVA. Several clinical studies have investigated the change in visual acuity in patients who underwent ATE-CXL. Improvement in CDVA was observed by Aixinjueluo et al. using ATE-CXL protocol at 30 mW/cm^2^ (22), but no change in CDVA was seen by Kir et al. (11) 2 years after treatment at 45 mW/cm^2^. Another study using ATE-CXL at 45 mW/cm^2^ reported a significant improvement in CDVA after 1 month, with no further change observed after 12 months (15). We also observed a decrease in SE from −6.96 ± 5.12 D at baseline to −4.67 ± 5.40 D at the last follow-up, but both had large margins of error (5.12 and 5.40 SD, respectively), resulting in no statistically significance difference between the two values. The improvement in UDVA in our study may be attributed to the decrease in SE.

No significant changes were observed in keratometry values, including astigmatism and maximum keratometry values. The finding that patients with PCE ≥ 80 μm tended to have decreasing post-operative Kavg and PCE suggests that advanced cases of progressive keratoconus might achieve pronounced stability after ATE-CXL. A previous investigation showed significant decreases in topographic parameters, including Kflattest, Ksteepest, and Kmax values, demonstrating that corneal CXL has a regularizing effect on the corneal shape (23). A significant decrease in Kmax was observed by Aixinjueluo et al. 12 months after ATE-CXL (22). Conversely, other studies showed no statistically significant differences in keratometry values (15, 24). Greater degrees of flattening have been described in patients with steeper corneas, as defined by Kmax values of more than 54.0 D (25). A comparable study also found significant flattening induced by CXL in an advanced subgroup with a Kmax value of more than 58.0 D (26), and another study demonstrated an improved CXL effect in early course of keratoconus patients with mean central K no more than 53 D (27). The reason for flattening of the K value may be that the corneal stroma in advanced cases has different arrangements of collagen. Another possibility is that CXL treatment might reach a deeper location in stroma in advanced cases, where the cornea is thinner and more elevated. Further study would be required to verify these speculations and to characterize the reasons why advanced keratoconus showed greater topographic improvement after ATE-CXL.

Corneal edema, post-operative dehydration, and redistribution during epithelial healing are common early after CXL procedures. Stable TCT was observed during our 4 years of observation. However, previous studies have reported conflicting results regarding change in corneal thickness. Following transepithelial 45 mW/cm^2^ CXL, one study found a significantly higher TCT value after 1 and 2 years (11), while another study found no significant change in central corneal thickness (CCT), but observed a significant decrease in TCT occurring between 6 and 12 months post-operatively (22). This change in corneal thickness might be attributed to the lamellar remodeling of the corneal stroma (28). In our study, patients with pre-operative TCT ≤ 450 μm showed a decrease in post-operative Kavg value and an increase in post-operative TCT. The mean pre-operative TCT was 491.90 μm in mild to moderate cases with Kmax lower than 58 D, and average change of Kmax after 4 years was elevated by 0.14 D. In advanced cases with Kmax above 58 D, the mean pre-operative TCT was 439.38 μm, and average change of Kmax after 4 years was reduced by 0.14 D. This indicates that advanced cases of progressive keratoconus with thin corneal thickness and high PCE could benefit from ATE-CXL and increase their chances of halting keratoconus progression.

Our study has several advantages. First, strict recruitment of progressive keratoconus patients was established; patients with a refractive surgery history and younger than 17 years of age, or with other systemic diseases were excluded. Second, ATE-CXL was conducted by the same experienced doctor with decades of surgical experiences (X. Zhou). Follow-ups were also performed by the same two occupational therapists (X. Zhang and L. Sun), and consistency was validated by an arbitrator (X. Zhou) to improve the accuracy of clinical information collection and assessment. Finally, our follow-up post-operative examinations provided detailed and granular insights. The LOCF method was used to ensure integrity of the last visit.

Our study was limited by the relatively small number of patients and the lack of a control group undergoing standard CXL, which may have increased the risk of bias. However, as a pilot study of the long-term outcomes of ATE-CXL, the aim of our study was to establish the clinical efficacy of this treatment by assessing the stability of progressive keratoconus, which our findings support. Additional randomized controlled trials with a larger sample size using several surgical protocols will be required to verify the impact of our procedure on keratoconus stabilization.

In conclusion, our study demonstrated that stabilization of progressive keratoconus can be achieved using ATE-CXL with irradiance of 45 mW/cm^2^ and 7.2 J/cm^2^. This procedure should yield better comfort during surgery and reduce complications for keratoconus patients. Advanced cases of progressive keratoconus might also achieve stability after ATE-CXL. However, the clinical efficacy of ATE-CXL in patients with thin corneal thickness and high PCE value will require more investigation.

**Precis:** ATE-CXL yields long-term stable outcomes in progressive keratoconus patients.

## Data Availability Statement

The datasets generated for this study are available on request to the corresponding author.

## Ethics Statement

The studies involving human participants were reviewed and approved by the Ethics Committee of the Eye and ENT Hospital of Fudan University. The patients/participants provided their written informed consent to participate in this study.

## Author Contributions

XZha, LS, and JZ were responsible for the initial plan, study design, data collection, data extraction, data interpretation, manuscript drafting, statistical analysis, and conducting the study. ML, MT, and YS were responsible for data collection, extraction, and critical revisions of the manuscript. XZha and XZho were responsible for data interpretation, manuscript drafting, supervision, and critical revisions of the manuscript for important intellectual content. This article's contents were solely the responsibility of the authors. XZho was the guarantor for this article and has full responsibility for this study. All authors contributed to the article and approved the submitted version.

## Conflict of Interest

The authors declare that the research was conducted in the absence of any commercial or financial relationships that could be construed as a potential conflict of interest.
